# Sex-dependent effects of psychedelics: review of evidence from rodent models

**DOI:** 10.3389/fpsyt.2026.1824073

**Published:** 2026-07-15

**Authors:** Rafał Marecki, Wiktoria Zaniewska, Adam Hamed, Napoleon Waszkiewicz, Jacob J. Baker, Kacper Łukasiewicz

**Affiliations:** 1 Department of Psychiatry, Medical University of Białystok, Białystok, Poland; 2 Laboratory of Spatial Memory, Nencki Institute of Experimental Biology, Warsaw, Poland; 3 Department of Molecular, Cell and Developmental Biology, University of California, Santa Cruz, Santa Cruz, CA, United States; 4 Experimental Medicine Centre, Medical University of Białystok, Białystok, Poland; 5 School of Human Sciences, VIZJA University, Warsaw, Poland

**Keywords:** 5-HT2A, dimethyltryptamine (DMT), lysergic acid diethylamide, psilocybin, psychedelic, rodents, sex differences

## Abstract

Classic psychedelics, including psilocybin, LSD, DMT, 5-MeO-DMT, mescaline, and phenethylamines such as DOI, exert their core effects through 5-HT2A receptor agonism, inducing widespread neurobiological and behavioral changes relevant to psychiatric research. Emerging evidence suggests that these effects are modulated by sex, yet this dimension remains underrepresented in studies. This review synthesizes findings from rodent research to examine sex-dependent variability across pharmacokinetics, physiology, neuroplasticity, behavior, and disease models. Notable sex differences appear in pharmacodynamics and neurobiological responses, such as divergent serotonergic and dopaminergic signaling patterns, sex specific central amygdala reactivity to psilocin, and differential patterns of dendritic spine formation following psilocybin administration. Behavioral outcomes - including head twitch responses, locomotor activity, prepulse inhibition, stress reactivity, and social behavior - frequently show stronger or qualitatively distinct effects in females than in males, with ovarian cycle phase further modulating several responses. Disease model studies similarly reveal divergent therapeutic or adverse outcomes, such as sex dependent effects of psilocybin on alcohol consumption and of DMT microdosing on affective behavior and neuroplasticity. Collectively, the evidence demonstrates that sex is a critical biological variable shaping the neural, physiological, and behavioral effects of psychedelics in rodents. Integrating sex specific analyses into experimental design is essential for improving translational validity and guiding clinical applications that account for biological differences in treatment response.

## 1 Introduction

Psychedelics are psychoactive compounds with a long history of human use, known for their effects on perception, emotional states, and thought patterns (1). Over the recent years, there has been increasing social attention on psychedelics, propelled by their therapeutic applications for mental health disorders like depression, anxiety, post-traumatic stress disorder (PTSD), or substance use disorders (2, 3). Psilocybin, lysergic acid diethylamide (LSD), dimethyltryptamine (DMT), 5-methoxy-N, N-dimethyltryptamine (5-MeO-DMT), mescaline, and 2,5-dimethoxy-4-iodoamphetamine (DOI) are often referred to as “classic psychedelics”. These substances primarily exert their effects through the serotonin system by acting as agonists or partial agonists at the 5-HT2A (5-hydroxytryptamine 2A) receptor, as both their hallucinogenic properties and therapeutic potential are primarily attributed to activation of this receptor. Pharmacological blockade or genetic knockout of 5-HT2A receptors substantially attenuates the characteristic psychedelic-induced alterations in perception and mood, as well as many of the downstream neuroplastic and affective changes associated with antidepressant and anxiolytic efficacy (4); however, evidence for 5-HT2A-independent contributions to therapeutic outcomes continues to emerge (5, 6).

Psychedelics can be systematically classified into distinct structural categories based on their core chemical scaffolds and biosynthetic origins. Tryptamines, such as psilocybin (O-phosphoryl-4-hydroxy-N, N-dimethyltryptamine), DMT (N, N-dimethyltryptamine), 5-MeO-DMT (5-methoxy-N, N-dimethyltryptamine), and 4-OH-DiPT (4-hydroxy-N, N-diisopropyltryptamine), are indoleethylamine derivatives biosynthesized from tryptophan and structurally analogous to endogenous serotonin (7). Their characteristic indole ring and ethylamine side chain enable extensive chemical variation. Phenethylamines, such as DOI (2,5-dimethoxy-4-iodoamphetamine), 25I-NBOMe (2-(4-iodo-2,5-dimethoxyphenyl)-N-[(2-(methoxyphenyl)methyl]ethanamine), and 2C-B (4-bromo-2,5-dimethoxyphenethylamine), are synthesized from phenylalanine and are based upon a phenyl ring. Linked to an ethylamine backbone; substitutions on the aromatic ring yield a broad spectrum of psychoactive effects (8). Additionally, lysergic acid diethylamide (LSD), a representative of the ergoline subclass of tryptamines, possesses a rigid tetracyclic structure combining elements of both indole and phenethylamine systems, and originates from ergot alkaloid biosynthesis.

Other compounds that can exhibit psychedelic-like effects include dissociatives, entactogens, deliriants, or oneirogens (7), ketamine, 3,4-methylenedioxymethamphetamine (MDMA), and salvinorin (9). All these substances produce their effects through various mechanisms, usually involving other neurotransmitter systems than classic psychedelics, such as glutamatergic, GABAergic, dopaminergic, or opioidergic (10).

Sex as a biological variable is increasingly acknowledged as a significant factor in biomedical research, and its role in shaping the effects of psychedelics might be crucial for understanding therapeutic potential and variability in outcomes (11–13). Tylš et al., in their work from 2016, highlight the critical role of sex steroids as key modulators of the effects of psychedelics (14). The influence of sex and gender on the subjective effects and pharmacological responses to various psychoactive drugs is profound (15). The persistent under-representation of female subjects in preclinical psychedelic research is reflected in the fact that most foundational rodent studies were conducted exclusively in males, leaving sex-dependent neuroplastic and pharmacological effects largely uncharacterized. A recent pig study reporting psilocybin-induced increases in SV2A density and reductions in 5-HT2AR density was conducted exclusively in females (16), itself highlighting how single-sex designs, regardless of which sex is chosen, leave the question of sex-dependent translational relevance unanswered. Sex differences in serotonin and tryptophan metabolism influence anxiety; specifically, 5-HT1A and 5-HT2C receptor expression, serotonin transporter (SERT) activity, and central serotonin levels each show sex-dependent variation that may modulate anxiogenic and anxiolytic responses (17). Notably, sex steroids such as estrogen, progesterone, and testosterone shape serotonergic function (17), which may also mediate sex-based differences in the effects of psychedelics. For example, researchers have observed sex-dependent differences in the effects of psychedelics, such as heightened emotionality and reduced tiredness in males versus more pronounced entactogenic effects in females under 2C-B (18).

Despite their translational limitations, rodent models remain essential for dissecting the neurobiological and pharmacological mechanisms underlying psychedelic effects, including the role of biological sex (2, 19, 20). Beyond subjective experiences in human studies, physiological responses to psychedelics also exhibit sex-based variations in animal models. For instance, Miliano et al. demonstrated that male and female rats exhibit distinct physiological reactions, including core temperature and analgesic effects, to NBOMe compounds, indicating that the toxicity and impact of this group of substances can vary between sexes (21). To better examine sex-dependent effects of psychedelics and their therapeutic value, we still need to leverage animal models, which, despite limitations, are essential for dissecting intricate neurobiological and pharmacological mechanisms. Rodents play a crucial role in understanding the complex models of pathophysiology and pharmacology of neuropsychiatric disorders (22). To better understand the effects of psychedelics, it is important to consider both intra-individual and inter-population variability within experimental animal groups. Rodent models offer a unique opportunity to systematically explore these variations, providing precise control over genetic, environmental, and experimental conditions.

This systematic exploration of variability in newly emerging psychedelic research is particularly crucial given the historical biases in biomedical research that have frequently neglected the significance of sex as a biological variable. Various researchers have noted that biomedical research underrepresents female subjects, particularly following the discovery of variability in the rodent estrous cycle (23). Studies of both sexes also often fail to analyze results by sex. Moreover, male bias was most prominently evident in neuroscience, where single-sex studies of male animals outnumber those of females in a 5.5 to 1 proportion (23). Underrepresentation of females in animal disease models is also commonplace, and our understanding of female biology remains compromised by these deficiencies (23, 24). Addressing this gap can give a perspective on how all factors - biological sex, strain, and individual behavioral phenotypes - modulate the effects of psychedelic substances in rodents, thus helping to uncover the mechanisms underlying variability in drug responses that are difficult to measure in human studies.

### 1.1 Background

#### 1.1.1 Molecular mechanisms of psychedelic action

Classic psychedelics are primarily classified based on their specific agonistic activity at serotonin 5-HT2A receptors (25, 26) - metabotropic receptors which, upon activation by a ligand (e.g., a psychedelic), functionally couple to the Gq/11 family of G proteins (27). A transient physical interaction with Gq/11 occurs when a ligand activates the receptor, initiating a downstream signaling cascade. Alternatively, the receptor may also engage the β-arrestin-2 pathway, wherein the adaptor protein β-arrestin regulates receptor internalization and serves as a signaling role by scaffolding kinases such as Raf, MEK, and ERK. This phenomenon, known as biased signaling, implies that different ligands can preferentially activate one of these pathways (Gq/11 or β-arrestin), leading to distinct cellular effects (28).

5-HT2A receptors are distributed, among other locations, on layer Va pyramidal neurons in the cortex and within the striatum (4, 27). These receptors modulate various cognitive, perceptual, and emotional processes, such as learning, memory, attention, creativity, mood, and consciousness (2). Experimental data suggest that only a small subset (~5%) of excitatory 5-HT2A-expressing neurons is directly activated following psychedelic administration, triggering secondary recruitment of specific GABAergic interneuron subtypes - including somatostatin-expressing cells in the somatosensory cortex and parvalbumin-positive cells in the medial prefrontal cortex (mPFC) - as well as astrocytes (~10%) (27). This leads to regionally heterogeneous network responses dependent on local microcircuit architecture (27). Upon receptor activation, the canonical Gq/11 → PLC → IP3/DAG → Ca2+ signaling cascade is initiated, leading to protein kinase C (PKC) activation and stimulation of the ERK/MAPK pathway, which in turn phosphorylates the transcription factor CREB (cAMP response element-binding protein) and enhances the expression of plasticity-related genes such as BDNF (brain-derived neurotrophic factor) (28). A key element of this cascade is glutamate: psychedelics increase cortical glutamate release, which activates NMDA (N-methyl-D-aspartate) receptors and facilitates CREB-dependent BDNF expression and TrkB activation (26, 29, 30). Psychedelics have also been proposed to exert an allosteric effect at TrkB, enhancing the activity of endogenous BDNF at active synapses (31), though this has not been consistently replicated (32).

Recent findings suggest that the efficacy of Gq/11 pathway activation is correlated with the magnitude of psychedelic behavioral effects, such as the head-twitch response in rodents (33) In contrast, the β-arrestin pathway - although engaged by some ligands - does not elicit typical psychedelic behaviors. Moreover, ligands biased toward β-arrestin signaling may promote receptor desensitization and internalization (34). This difference in signaling efficacy, particularly the degree of Gq/11 pathway activation, may explain why compounds with similar receptor binding profiles exhibit varying psychedelic potency (34, 35).

The functional consequences of 5-HT2A activation extend beyond neuronal excitation to network-level changes, including local cortical desynchronization, disintegration of the default mode network (DMN), increased signal variability, and reorganization of functional connectivity between sensory and associative regions (36). Classic psychedelics also function as psychoplastogens - compounds that induce rapid and lasting structural plasticity, including increased dendritic spine density and the formation of new synapses in the cortex. These effects have been linked to the BDNF–TrkB signaling pathway and structural plasticity in the adult brain (37, 38), though direct evidence for TrkB-mediated reopening of critical periods specifically remains limited.

While 5-HT2A is the primary pharmacological target of classical psychedelics, individual compounds differ in their receptor binding profiles. For instance, LSD acts on 5-HT2A, 5-HT2B, 5-HT2C, and 5-HT1A receptors with varying affinities, as well as on dopamine D1–D3 receptors (39). Additionally, it binds to adrenergic α1A and α2A receptors, which may contribute to autonomic effects such as changes in blood pressure, thermoregulation, and arousal (39, 40). Mescaline binds to 5-HT2A, 5-HT1A, and adrenergic α2A receptors. Psilocin, the active metabolite of psilocybin, primarily stimulates 5-HT2A, 5-HT1A, and 5-HT2C receptors, contributing to anxiolytic and antidepressant effects by modulating serotonin release and downstream signaling pathways. Glutamatergic signaling is also strongly affected by psilocybin (41).

Individual differences in psychedelic response may partially stem from genetic variants in the HTR2A gene encoding the 5-HT2A receptor. The most studied polymorphisms - rs6311 in the promoter region and rs6313 in the exon may influence receptor expression. However, evidence that these variants explain sex-dependent differences is limited. Sex differences in HTR2A allele frequencies are not prominent in most populations, although one Iranian study reported a higher frequency of the rs6313 C allele in women (42). Moreover, meta-analytic evidence suggests that rs6311 and rs6313 alone do not significantly predict depression risk, indicating that their functional impact may depend on hormonal, epigenetic, or gene-gene interactions (43).

More relevant to sex differences in psychedelic action may be hormonally driven regulation of the serotonergic system. Estradiol increases the expression of 5-HT2A and tryptophan hydroxylase 2 (TPH2), the rate-limiting enzyme in central serotonin synthesis (44, 45). PET studies indicate that baseline 5-HT2A binding potential does not differ significantly between men and women, but estradiol levels positively correlate with receptor density (46).

Furthermore, the timing of psychedelic administration intersects with cyclical fluctuations in gonadal steroids. Evidence from preclinical studies indicates that the capacity for synaptic remodeling varies across the estrous cycle, with estrogen promoting spinogenesis and synaptogenesis in the hippocampus and prefrontal cortex via both genomic (ERα, ERβ) and rapid nongenomic (membrane ERs, GPER1) mechanisms (47).

#### 1.1.2 Behavioral response to psychedelics in rodents

Primary methods in researching psychedelics using animal models are drug discrimination tests and head-twitch response tests (HTR), which align remarkably well with their hallucinogenic potencies in humans, evaluated across a range of distinct molecules (33). Drug discrimination tests evaluate the discriminative stimulus properties of psychoactive drugs (48). The head-twitch response (HTR), a rapid side-to-side head movement and a component of the serotonin behavioral syndrome, is most readily elicited in mice, less consistently in rats and rabbits, and in rats often appears as “wet dog shakes” involving the head, neck, and trunk (33). While the expression of HTR can be regulated or modulated by multiple receptors and neurotransmitter systems, numerous studies have confirmed that this behavior is associated explicitly with 5-HT2A activation (33). One main difference between these tests is that the HTR is an innate behavior, an unconditional response, while in the drug discrimination tests animals are trained to distinguish the internal effects of a psychoactive drug from a non-drug state (48).

Psychedelics elicit a wide array of behavioral effects in animal models. Beyond drug discrimination tests and HTR, several other behavioral paradigms have been explored to capture the diverse effects of psychedelic drugs. For instance, changes in locomotor activity are a hallmark of psychedelic administration in rodents, primarily mediated by 5-HT2A receptor activation (33, 49, 50). Low doses of mescaline and 2,5-dimethoxy-4-methylamphetamine (DOM) increase locomotion, while higher doses reduce it (49, 51). Psilocin generally suppresses activity but can have dose-dependent effects, initially impairing movement at 4 mg/kg before increasing it, whereas 1 mg/kg has no significant impact (52). LSD disrupts stereotyped locomotor patterns in novel environments, maintaining variability and novelty-driven exploration throughout testing (53).

Classic psychedelics have been shown to disrupt prepulse inhibition (PPI), a measure of sensorimotor gating, mainly through 5-HT2A-dependent mechanisms. In 129S6/SvEv mice, it was demonstrated that DOI increased PPI in a 5-HT2AR-dependent manner, exhibiting different effects on startle amplitude in males versus females (54). In a different study done by Ouagazzal et al., for both Sprague-Dawley and Wistar rats, LSD caused disrupted PPI and induced a range of behaviors consistent with 5-HT receptor activation. Crucially, the disruption of PPI by LSD was reversed only by a selective 5-HT2A antagonist and not by 5-HT2C, 5-HT1A, 5-HT6, or D2 antagonists (55). The attenuation of PPI is considered a behavioral correlate of altered sensory processing, which parallels some aspects of psychedelic-induced perceptual changes also in humans.

Some behaviors are linked to the actions of classic psychedelics, although they cannot be considered direct indicators. Vertical exploratory movements, or rearing, are often studied in psychedelics research (51, 56). Increased rearing may signify an augmented propensity for novelty-seeking behavior, a phenomenon that could be pertinent to comprehending the influence of psychedelics on exploratory tendencies (51). Self-grooming is another behavioral response studied in this context (57). For instance, in the SAPAP3 knockout mouse model of obsessive-compulsive disorder-like pathology, characterized by excessive self-grooming, psilocybin significantly attenuated self-grooming behaviors compared to vehicle-treated controls. A single administration of either psilocybin or mushroom extract containing psilocybin (PME) reduced grooming duration and bouts, with effects persisting up to six weeks (57).

Social behavior is another important area of study, although conflicting data exists regarding social interactions and social contact markers in rodents following psychedelic administration (58, 59). However, in some cases, psychedelics can transiently diminish social behavior, reflecting altered sensory processing or heightened environmental sensitivity. In this context, another factor worth examining is ultrasonic vocalizations (USVs), which are closely linked to neurotransmitter dynamics within the ascending pathways of the mesolimbic dopaminergic and cholinergic systems (60). This provides a noninvasive and real-time indicator of their activity in the limbic region. Current evidence on the effects of psychedelics on ultrasonic vocalizations is limited. In the study of Jefferson et al., 5-MeO-DMT (20 mg/kg) caused substantial suppression of social ultrasonic vocalizations produced during mating behavior (61), with females vocalizing less frequently. Given their sensitivity to many psychoactive compounds, including those targeting affective and motivational processes, USVs have been widely employed in neuropharmacological research to assess drug-induced changes in emotional states and social behaviors (62). Ultrasonic vocalizations (USVs) are an underexplored marker indicating neurochemical dynamics in psychedelic-induced changes in social and emotional processing.

Psychedelic substances have demonstrated promising results in the treatment of various mental disorders. Comprehending sex-specific differences in disease pathology necessitates the recognition that these distinctions may originate from a multitude of factors, including both molecular mechanisms and more complex processes associated with biological sex. Accordingly, we have chosen to examine psychedelic research that employs diverse experimental models, moving beyond simplistic attributions to hormonal variation alone.

### 1.2 Aims of this review

This review synthesizes evidence on sex-dependent effects of psychedelic compounds in rodent models. Findings are organized by level of analysis, beginning with pharmacokinetic and physiological differences, followed by neuroplasticity, then unconditional and conditional behavioral responses, and finally outcomes in rodent models of psychiatric disorders.

The historical “male bias” in preclinical research has distorted our understanding of drug responses and created gaps in treatments for female biology. Addressing these biases by including male and female subjects in rodent studies can offer a critical perspective on the neurobiological and behavioral potential of psychedelics. This is essential for developing translational treatments that consider the needs of both sexes.

## 2 Methodology

To optimize our review, on 27.01.2026, we used a scraping script run in a Python environment to create a database of all publications addressing classic psychedelics, including keywords: “ayahuasca”, “dimethyltryptamine”, “lysergic acid diethylamide”, “mescaline”, “psilocybin” (**Supplementary 1**). A scraping script is a programmed tool that automatically extracts and compiles data from online sources, such as PubMed, by searching for specific keywords and retrieving relevant publications. The output consisted of seven lists, totaling 12,320 publications. We then excluded publications in languages other than English, which resulted in a further reduction to 10,986. After removing duplicates, the amount was reduced to 10,263.

In our next step, we sorted the database using the following phrases: “female”, “male”, “mice”, “rat”, “gender”, “sex”, “sex-dependent”, “sex differences”, and “sex-specific” (**Supplementary 2**). Each paper was assigned with points based on keywords appearing in abstract from the first and the second list. One point was an equivalent of true value for one keyword.

The models we focused on were designed for rodents. During our research, we came across papers covering sex differences in the context of psychedelic substances not included in the original query: ibogaine, DOI, 5-MeO-DMT, 4-OH-DiPT, 25I-NBOMe, and 2C-B. We included these additional compounds for several reasons. All of them show a strong affinity for the 5-HT2A receptor, the main target of classic psychedelics, making them mechanistically relevant. These compounds are also widely used in preclinical research to probe serotonergic function and plasticity, offering potential insights into sex-specific effects of psychedelics. Excluding them would have meant overlooking key findings from adjacent pharmacological domains that are essential for understanding how sex modulates psychedelic action in animal models.

After browsing all matching publications, we chose twenty-one core papers, which in our opinion clearly show sex dependent effects of psychedelic compounds. The earliest came from 1986 and the latest from 2025, as we did not use the time of publication criteria. Different strains of mice and rats were used across the selected studies, which we also find necessary to mention. The studies included a range of rodent strains, including C57BL/6J and 129S6/SvEv mice, as well as Sprague–Dawley, Long Evans, Wistar, and Fawn Hooded rats (**Supplementary 3***)*.

We organized the findings into two subsections that move from molecular and physiological substrates toward whole-animal output. Section 3.1 (Pharmacology, Physiology, and Neuroplasticity) synthesizes sex-dependent differences in pharmacokinetics (drug distribution, brain and plasma concentrations, elimination), in physiological responses (locomotor and sensorimotor function, body temperature, nociception), and in structural and molecular plasticity (dendritic spine dynamics, gene expression, receptor signaling). Section 3.2 (Behavioral effects) covers unconditional reflexive responses in 3.2.1 (head-twitch, prepulse inhibition, locomotion), learned context-dependent behavior in 3.2.2., and disease-model paradigms in 3.2.3. relevant to depression, anxiety, addiction, and OCD. Where applicable, we report estrous-cycle phase as a separate variable, given its demonstrated influence on whether sex differences emerge.

## 3 Results

### 3.1 Pharmacology, physiology and neuroplasticity

One key study directly addressing the pharmacological model of psychedelic action was identified, in which volinanserin, a 5-HT2A receptor antagonist, completely blocked DOI-induced HTR in both male and female C57BL/6J mice (63). Notably, the brain rapidly takes up DOI, leading to fast-onset pharmacodynamic effects, which is a crucial factor when interpreting behavioral responses. Inositol monophosphate (IP1) accumulation in the frontal cortex following DOI administration did not show any sex-related effect in C57BL/6J mice (63). However, the pharmacokinetic properties of DOI varied between sexes. Brain and plasma concentrations of DOI were lower 30 and 60 min after drug administration in female compared to male mice (63).

We have found two relevant rodent studies that used psychedelics that revealed insights into how these compounds can shape physiology and neuroplasticity, in ways that significantly differ between sexes. This number is still limited due to the very limited number of studies conducted with psychedelics. 25I-NBOMe, also known as “N-Bomb,” is a synthetic phenethylamine that produces psychedelic and entactogenic effects. *In vivo* microdialysis was used to assess the impact of 25I-NBOMe on dopaminergic (DA) and serotonergic (5-HT) transmission in the nucleus accumbens (NAc) shell and core, as well as the medial prefrontal cortex (mPFC) of male and female rats (21). Female rats exhibited higher dopamine (DA) levels in the medial prefrontal cortex (mPFC), whereas males showed a stronger serotonin (5-HT) response in this region (21). DA levels also increased in the NAc shell in both sexes, though with distinct temporal patterns, while the NAc core effect was exclusive to males. It also impaired visual responses in both sexes, significantly affected core temperature in females, and exhibited an analgesic effect in male rats at the highest tested dose (21). The drug also impaired the startle amplitude equally in both sexes and inhibited PPI in both male and female rats (21).

Psilocybin and its active metabolite psilocin act rapidly and show long-lasting symptom improvements in a variety of psychiatric disorders (64–67). Among the brain regions underlying these effects, the central amygdala (CeA) a primary output of the extended amygdala dysregulated in stress-, fear-, and anxiety-related disorders, has been identified as a key site of sex-specific reactivity. In Effinger et al. (2023), CeA reactivity was assessed using c-Fos as a marker in fiber photometry paired with an aversive air-puff stimulus (13). Results show that psilocin administration acutely increased the amygdala’s activity in both sexes, while increasing stimulus-specific CeA reactivity in females, but not males. In contrast, psilocin produced time-dependent decreases in reactivity in males, but not in females, only 2 days post-administration, lasting up to 28 days (13). They also measured behavioral responses to the air-puff stimulus and found that psilocin reduced threat responding more prominently in males than in females (13). Repeated presentations of the auditory component were also performed, revealing greater psilocin-induced suppression of CeA reactivity to the auditory-alone stimulus in males compared to females (13). (**Table 1**)

**Table 1:** Pharmacology, vegetative functions, and brain physiology.

Study	Subjects	Drug	Findings
Jaster et al., (63)	C57BL/6J mice 129S6/SvEv	DOI	5-HT2AR antagonist blocked HTR (DOI induced) equally in C57BL/6J males and females. No sex-related effect on IP1 accumulation. Lower brain and plasma DOI concentrations in females, with comparable or heightened HTR rates.
Miliano et al., (21)	Sprague–Dawley rats	25I-NBOMe	Greater ↑ in mPFC dopamine levels after 25I-NBOMe in females. ↑ serotonergic response in the mPFC in male rats. Dopamine ↑ in the NAc shell in both sexes but with distinct temporal patterns; effect in the NAc core exclusive to males. 1.0 mg/kg did not elevate dopamine levels. 25I-NBOMe impaired visual responses and startle amplitude equally in both sexes; ↓ PPI in both males and females. Core body temperature ↓ only in females. Analgesic effect observed at high dose specifically in males.
Effinger et al., (13)	Sprague–Dawley rats	Psilocin	Psilocin ↑ CeA activity in both sexes. ↑ stimulus-specific CeA reactivity in females; Time-dependent ↓ in reactivity in males. Sex-dependent changes in threat responding; no changes in exploratory behavior or general locomotion. Sex-specific effects on CeA reactivity to auditory-alone stimulus.

Decreases and increases of particular values are noted respectively using symbols: (↓) and (↑). mPFC, medial prefrontal cortex; NAc, nucleus accumbens; PPI, prepulse inhibition; CeA, central nucleus of the amygdala 5HT2AR, serotonin receptor type 2A; HTR, head twitch response; DOI, 2,5-Dimethoxy-4-iodoamphetamine; IP1, inositol monophosphate.

Summary of the available evidence on sex-related differences in the pharmacodynamics of psychedelics and their effects on vegetative functions and brain activity/physiology, highlighting the substance used, species, and strain of tested animals.

To examine psilocybin-induced structural plasticity, Shao et al. (68) administered psilocybin to male and female C57BL/6J mice and tracked changes in dendritic spines in the medial frontal cortex using chronic two-photon microscopy. At the dose used (1 mg/kg, i.p.), psilocybin reliably elicited head-twitch responses, confirming pharmacological activity.

They compared psilocybin (1 mg/kg, i.p.) with saline and ketamine (10 mg/kg, i.p.), which served as negative and positive controls, respectively (68). Psilocybin led to a rapid and persistent increase in spine density and size, with structural changes observed within 24 hours and lasting at least one month (68). It also improved stress-related behavioral deficits. The spine formation rate in females increased by 8% ± 2% after psilocybin (68). Similarly, the spine formation rate was higher by 4% ± 2% in males after psilocybin (68). Replication in a separate cohort confirmed that psilocybin-induced increases in spine density in Cg1/M2, referring to cingulate cortex area 1 and secondary motor cortex, were more pronounced in females than in males, with similar female-biased effects observed in primary motor cortex (68). In contrast, there was no change in the elimination rate of spines (68). The increase in spine formation rate was highest shortly after psilocybin administration. It then gradually diminished in subsequent days, returning to a baseline level and reaching equilibrium with the elimination rate (68).

Villalba et al. (69) examined sex- and genotype-dependent effects of noribogaine (40 mg/kg, i.p.), the primary active metabolite of ibogaine, on locomotion, mPFC gene expression, and NMDA current density in 5-HT2AR wild-type (WT) and knockout (KO) mice. Noribogaine reduced locomotor activity in all male mice regardless of genotype, indicating a 5-HT2A-independent hypolocomotor mechanism in males. In females, this effect was observed only in KO mice, suggesting that intact 5-HT2AR signaling buffers against noribogaine-induced hypolocomotion in females. mPFC gene expression showed sex- and genotype-dependent patterns, with females generally responding at higher doses (40 mg/kg) and males at both doses tested. Noribogaine additionally reduced NMDA-mediated postsynaptic current density selectively in male WT mice, further supporting a sex-specific role of 5-HT2AR in modulating glutamatergic transmission in the mPFC (69).

In another study, a single dose of ibogaine (60 mg/kg) was administered to C57BL/6J mice, and frontal cortex gene expression was assessed 4 hours later (70). Transcriptomic analysis showed sex-dependent effects, with broader changes in females than in males. In the pooled comparison, seven genes differed between ibogaine and control groups: Oxt, Avp, Cbln2, and Cbln4 were upregulated, whereas Usp35, Ap5b1, and Gm34306 were downregulated. Sex-stratified analyses identified changes in eight genes in males and 28 genes in females, most with large effect sizes (70). qPCR validation confirmed ibogaine-related upregulation of Cbln4 and downregulation of Usp35, while females showed lower expression of Cbln2 and Cbln4 than males (70). Overall, these findings suggest that ibogaine produces stronger transcriptomic changes in females, particularly in genes related to synaptogenesis and neuropeptide signaling (70). (**Table 2**)

**Table 2:** Genes, receptors, and neuroplasticity.

Study	Subjects	Substance	Findings
Shao et al., (68)	C57BL/6J mice	Psilocybin, ketamine	Sharp rise in head-twitch responses at 1 mg/kg dose. Rapid and sustained increase in dendritic spine density and size. Improvement in stress-related behavioral deficits. Spine formation rate increased by 8% ± 2% in females. Spine formation rate increased by 4% ± 2% in males.
Villalba et al., (69)	129S6/SvEv 5-HT2AR knockout and wild type mice	Noribogaine	• Locomotion decreased in male WT/KO and female KO; no effect in WT females. • Male WT: ↑ 5-HT2AR, GRIN2A (10 mg/kg); NMDAR ↓ (40 mg/kg). • Female WT: ↑ Egr1, cFos, 5-HT2AR (40 mg/kg); no change in NMDAR. • Male KO: ↑ Npas4, Egr1 (10 mg/kg); ↑ cFos, GRIA1 (40 mg/kg); no change in NMDAR. • Female KO: ↑ Npas4, GRIN2A (10 mg/kg); no change in NMDAR.
Biosca-Brull et al., (70)	C57BL/6J mice	Ibogaine	• 4 genes upregulated (Oxt, Avp, Cbln2, Cbln4); 3 downregulated (Usp35, Ap5b1, Gm34306). • 8 gene changes in males, 28 in females. • Cbln2 and Cbln4 expression lower in females.

Decreases and increases of certain values are noted respectively using symbols: (↓) and (↑). WT, wild type; KO, 5-HT2AR knock-out; Npas4, neuronal PAS domain protein 4 gene; Egr1, early growth response 1 gene; cFos, protein c-Fos gene; GRIA1, glutamate receptor 1 gene; GRIN2A, glutamate receptor subunit epsilon-1 gene; HT2AR, serotonin receptor type 2A; NMDAR, N-methyl-D-aspartate receptor; Oxt, oxytocin gene; Avp, arginine vasopressin gene; Cbln2, cerebellin 2 precursor; Cbln4, cerebellin 4 precursor; Usp35, ubiquitin specific peptidase 35; Ap5b1, adaptor related protein complex 5 subunit beta 1; Gm34306, predicted gene 34306.

Summary of the available evidence of sex-related differences in effects of psychedelics on dendritic spines, genes, and receptor expression, highlighting the used substance, species, and strain of tested animals.

### 3.2 Behavioral effects

#### 3.2.1 Unconditional

Dickinson and Curzon (71) assessed behavioral responses to 5-MeO-DMT in male and female Sprague-Dawley rats. No significant sex differences were observed in any measured behavior following 5-MeO-DMT administration, including locomotion, sniffing, rearing, grooming, and gnawing. However, the Straub tail response, that is one component of the serotonin behavioral syndrome, was significantly higher during early diestrus than proestrus, indicating that estrous cycle phase modulates at least some aspects of 5-MeO-DMT-induced behavior in females (71).

In a study by Jaster, mentioned in the previous section, they examined the effects of DOI on head-twitch behavior – a mouse behavioral indicator of human psychedelic potential – in both male and female mice (63). They found that DOI induced more HTR in female than in male C57BL/6J mice, a sex-specific behavior that was not observed in 129S6/SvEv animals (63). What is essential is that there is a baseline strain difference in motor behavior (HTR before drug administration) (63).

Furthermore, a study assessed the effects of psilocybin (1 mg/kg) on decision-making and motivation in healthy rats of both sexes, using a head twitch response to confirm psilocybin activity (72). The authors measured how the rats chose between rewards that varied in probability and delay, as well as their effort exertion for rewards (72). This study showed that psilocybin did not alter decision-making or motivation in rats (72). However, as expected, it increased head twitches, confirming the drug’s effectiveness at the given dose (72). Additionally, the researchers found no significant sex differences in the proportion of large choices in probability and delay discounting tasks, nor in the rate of decay or area under the curve (72). They also observed no significant sex differences in cumulative responses or breakpoints in the progressive ratio task (72). These findings suggest that the therapeutic benefits of psilocybin are unlikely to result from alterations in brain systems responsible for reward and decision-making (72), and sex-related factors do not influence them.

Páleníček et al. (73) examined locomotor activity and prepulse inhibition (PPI) following LSD administration (5, 50, and 200 µg/kg, s.c.) in male rats and females stratified by estrous cycle phase: pre-ovulatory (EP: estrus/proestrus) and post-ovulatory (MD: metestrus/diestrus). LSD reduced locomotor activity in males and MD females, whereas EP females showed increased locomotion in the second half of testing. Both female groups exhibited less thigmotaxis and were less sensitive to LSD-induced hypolocomotion than males. PPI was disrupted in males and MD females but not in EP females, demonstrating that estrous cycle phase critically determines the direction and magnitude of LSD-induced behavioral effects (73).

Tylš et al. (14) investigated sex-dependent effects of psilocin (0.25, 1, and 4 mg/kg) on locomotion and sensorimotor gating in male and female Wistar rats, with females stratified by estrous cycle phase (EP: proestrus/estrus; MD: metestrus/diestrus). Males and MD females showed dose-dependent reductions in locomotor activity, while EP females were unaffected, mirroring the estrous cycle-dependent pattern observed with LSD. Females of both cycle phases spent more time in the arena center than males, and showed greater wet dog shakes at 0.25 mg/kg and more sniffing at 1 mg/kg. The male-biased suppression of locomotion and sensorimotor gating was partially reversed by 5-HT1A and 5-HT2B/C antagonists, but not by 5-HT2A blockade (14).

One of these crucial changes in the psychedelic-induced perception changes is the disruption of sensorimotor gating, which is the ability to filter out irrelevant sensory information. Sensorimotor gating is measured by prepulse inhibition (PPI) of the startle response, which is reduced in certain mental disorders (54). Vohra et al. (54) assessed the effects of DOI (0.5 mg/kg, i.p.) on startle and PPI in male and female 129S6/SvEv mice across varying acoustic stimuli. DOI increased PPI in males in three out of four conditions, an effect blocked by the 5-HT2AR antagonist volinanserin, confirming a 5-HT2AR-dependent mechanism. In females, DOI increased PPI without altering startle amplitude. Notably, PPI was positively correlated with startle in males but negatively in females, indicating a fundamental sex difference in sensorimotor gating. LSD similarly increased PPI in males without affecting startle (54).

Another study examining unconditional behavior investigated sex differences in psychedelic response through systematic behavioral assessment. A head twitch response, an elevated zero maze, and a probabilistic reversal learning test were performed. The research revealed sex-based differences in acute responses to psilocybin, particularly in adult subjects (74). Adult females exhibited significantly more head twitch responses (HTRs) compared to males when administered psilocybin, demonstrating enhanced sensitivity to the drug’s acute effects. This sex difference was further modulated by hormonal state, with females in diestrus showing increased HTR compared to those in proestrus (74). Interestingly, these behavioral differences were not reflected in baseline 5-HT2A receptor expression in the medial prefrontal cortex, which showed no significant sex differences in either adolescent or adult animals. The enhanced female sensitivity aligns with previous findings on other psychedelics like DOI (63). Despite these differences in acute response, the study found no significant sex differences in longer-term behavioral outcomes. Both males and females showed comparable performance in anxiety-associated behaviors on the elevated zero maze test following adolescent psilocybin exposure. Similarly, the probabilistic reversal learning task revealed no sex differences in either initial task acquisition or behavioral flexibility after reversal learning (74).

#### 3.2.2 Conditional

Meehan and Schechter (1998) investigated the effects of LSD on male and female Fawn Hooded rats (75). The study used a biased design, where the rats were given LSD (0.2 mg/kg, IP) and then confined to either their preferred or non-preferred location. Control rats were confined to both locations after being given saline (75). The results showed that rats given LSD and confined to the non-preferred location spent more time in that location during a non-drug test (75). This behavior, indicative of a conditioned place preference, was only observed in male rats (75). The authors suggest that the lack of conditioned place preference only in female Fawn Hooded rats may be due to methodological constraints, as the study did not control for estrous cycle stages (75). They hypothesize that fluctuations in estrogen levels, which influence serotonin metabolism, could have introduced variability that masked the conditioned drug effect in females (75). (**Table 3**)

**Table 3:** Unconditional and conditional behaviors.

Study	Subjects	Substance	Findings
Dickinson and Curzon (71)	Sprague–Dawley rats	PCA, 5-MeODMT	• Females had more pronounced tremor after PCA.
Jaster et al., (63)	C57BL6/J and 129S6/SvEv mice	DOI	• DOI induced more HTR in female C57BL/6J mice
Roberts et al., (72)	Long Evans rats	Psilocybin	• No change in decision-making or motivation, increased head twitches.
Páleníček et al., (73)	Wistar rats	LSD	• Locomotor activity ↓ Thigmotaxis ↑ and PPI disruption ↑ **in males**. • Locomotor activity ↓ (less pronounced than males) Thigmotaxis ↓ vs males PPI disruption ↑ in **MD females**. • Locomotor activity ↑ (second half of test) Thigmotaxis ↓ vs males. Rearing ↑ (after 5 µg/kg). No PPI disruption in **EP females**.
Tylš et al., (14)	Wistar rats	Psilocin	• Females less affected by locomotor inhibitory effects, ↑ wet dog shakes in females
Vohra et al., (54)	129S6/SvEv mice	DOI, LSD, apomorphine, SKF-82,958	• DOI ↑ PPI in females, LSD ↑ PPI in males
Zylko et al., (74)	Long Evans rats	Psilocybin	• Psilocybin ↑ HTR in females, especially in diestrus. • No sex differences in 5-HT2AR expression, anxiety, or reversal learning.
Meehan and Schechter (75)	Fawn Hooded rats	LSD	Rats given LSD and confined to non-preferred location spent more time there during non-drug test. Conditioned place preference observed only in male rats.

Decreases and increases of certain values are noted respectively using symbols: (↓) and (↑). PCA, para-chloroamphetamine; HTR, head-twitch response; PPI, prepulse inhibition; LSD, lysergic acid diethylamide; 5-HT2AR, serotonin receptor type 2A; 5-MeODMT, 5-methoxy-N,N-dimethyltryptamine; DOI, 2,5-dimethoxy-4-iodoamphetamine. EP females - females in the oestral and pro-oestral phase; MD females, females in the metaestrus and diestrus phase.

Summary of the available evidence on sex-related differences in the effects of psychedelics on unconditional and conditional behaviors, highlighting the substance used, species, and strain of tested animals. Bold values indicate findings in which a sex- or estrous-cycle-dependent difference was reported.

#### 3.2.3 Rodent models of psychiatric disorders

In a subsequent study, researchers used Sprague-Dawley rats to investigate the effects of restraint stress on behavior and serotonin metabolism (76). The impact of stress on behavior, serotonin metabolism, and responses to 5-MeO-DMT were compared between male and female rats (76) Kennett et al. The effects of restraint stress were assessed using behavioral measures, regional serotonin metabolism, and behavioral sensitivity to 5-MeO-DMT (76). Following a single restraint episode, females showed smaller stress-related changes in these measures than males (76). However, after repeated restraint, females did not show the same adaptive pattern observed in males, as reflected by persistent changes in 5-hydroxyindoleacetic acid concentrations in selected brain regions, particularly the frontal cortex (76). Female rats also did not show increased behavioral sensitivity to 5-MeO-DMT after repeated stress. Moreover, female rats, but not males, had lower concentrations of 5-hydroxyindoleacetic acid in specific brain regions, particularly the frontal cortex, when euthanized 24 hours after the final restraint period (76).

Psilocybin is being assessed as a potential treatment for alcohol use disorder (AUD) (77). In male C57BL/6J mice, alcohol consumption and preference decreased during the three days immediately after psilocybin administration, in a dose-dependent manner consistent across doses of 0.5 mg/kg or higher (77). In female mice, psilocybin did not significantly affect alcohol consumption or preference at any dose or time point (77), suggesting that psilocybin may be more effective at reducing alcohol intake in males than in females (77).

“Microdosing”, which means in the context of psychedelics intermittent chronic administration of low doses, was evaluated for potential beneficial effects on male and female Sprague Dawley rats using the psychedelic N, N-dimethyltryptamine (DMT) (78). The observed impact of this dosing regimen differed from that induced by a single high dose of the drug. The study found that low doses of DMT administered chronically and intermittently resulted in an antidepressant-like effect and improved fear extinction learning, without affecting working memory or social interaction (78). In terms of dendritic spine density, there was a significant reduction in the number of spines per 10 μm on layer V pyramidal neurons in the PFC in female rats treated with DMT, but no such change was observed in male rats (78). Furthermore, male rats administered DMT showed a notable increase in body weight compared to controls, while female body weight remained unchanged (78). The authors attribute this to prolonged excitotoxicity related to increased glutamate release. The absence of this effect in females, alongside the observed reduction in dendritic spine density in females but not males, may suggest that females are more susceptible to DMT-induced excitotoxic effects on prefrontal circuitry (78).

Kelly et al. investigated the effects of 4-OH-DiPT on fear extinction learning in male and female mice (79). 4-OH-DiPT significantly decreased freezing responses to conditioned cues in a dose-dependent manner, with higher potency observed in female mice compared to male mice (79). Female mice that were given 4-OH-DiPT before extinction training showed reduced avoidance behaviors in the light-dark box, elevated plus maze, and novelty-suppressed feeding test several days later compared to controls. However, male mice did not show significant differences (79).

With a rapidly growing interest in the use of psychedelics for treating many mood disorders, not a lot is being discussed about their impact on post-acute mood-altering actions (80). In a recent study, adult male and female C57BL/6J mice were given saline or psilocybin (5 mg/kg, i.p.) and underwent a variety of tests. Psilocybin administration significantly increased the frequency of head-twitch responses (HTRs) in both sexes, with females exhibiting a more pronounced HTR effect over the 30-minute trial, despite no baseline differences. It has been shown that psilocybin acutely increases HTR frequency, with greater effects observed in females (80). After 24h post-injection, Farinha-Ferreira et al. examined the interaction between psilocybin (5 mg/kg, i.p.) and acute restraint anxiety stress- and depressive-like behaviors in tested animals. When administered under controlled conditions, psilocybin significantly reduced negative emotionality, with pronounced effects observed in the marble burying test (MBT) and the novelty-suppressed feeding test (NSFT). In male mice acute stress exposure completely abrogated the anxiolytic effects of psilocybin. Female mice demonstrated a more nuanced response, with psilocybin maintaining partial anxiolytic effects even under stressful conditions (80). Lastly, neuroendocrine analysis revealed sex-specific corticosterone responses: in males, stress alone and stress combined with psilocybin produced the highest corticosterone elevations, while psilocybin under non-stress conditions also significantly raised corticosterone levels. In females, corticosterone was similarly elevated across all conditions: psilocybin alone, stress alone, and their combination, with no condition producing a markedly higher response than others (80).

Chronic psilocybin effects were also evaluated in wild-type and SAPAP3 knockout mice, a preclinical model of OCD-like phenotypes (81). The main genotype-related finding was impaired sociability in SAPAP3 knockout mice. Psilocybin produced sex-dependent effects primarily in wild-type animals: it increased sociability selectively in male wild-type mice and altered specific microbiota taxa only in this group (81). Across groups, chronic oral psilocybin increased gut transit time in a dose-dependent manner, while microbiome diversity remained broadly unchanged (81). Integrative analyses linked a Lactobacillus/Alistipes microbial cluster to locomotion, head-twitch response, and gut transit measures, suggesting a microbiota-gut-brain mechanism influenced by sex and genotype (81).

A recent multi-lab preprint evaluated psilocybin (2 mg/kg, i.p.) in adult male and female C57BL/6J mice across five independent laboratories, separating acute from 24-h post-dose outcomes (82). Psilocybin produced highly replicable acute effects, most notably robust head-twitch responses (tending to be higher in females) and consistent reductions in anxiety/avoidance-like measures in assays such as the open field and elevated plus maze, alongside reduced fear expression during retrieval (82). By contrast, researchers found little evidence for reliable 24-h “persistent” behavioral effects across sites (including depression-like measures, anxiety-like behavior, social preference, or consistent facilitation of fear extinction), suggesting that in healthy mice many purported longer-lasting effects may be small and/or strongly context-dependent (82) (**Table 4**).

**Table 4:** Animal models.

Study	Subjects	Substance	Findings
Kennett et al., (76)	Sprague-Dawley rats	5-MeODMT	Male rats: decreased movement, increased defecation after single stress; adaptation to repeated stress. Female rats: less impacted by single stress, no adaptation to repeated stress.
Alper et al., (77)	C57BL/6J mice	Psilocybin	Male mice: decreased alcohol consumption and preference. Female mice: no significant effect.
Cameron et al., (78)	Sprague Dawley rats	DMT	Antidepressant-like effect, improved fear extinction learning, increased body weight in males. Decrease in the dendritic spine density in the frontal cortex of female, but not male rats.
Kelly et al., (79)	*C57BL/6J mice* Human 5-HT GPCRs	4-OH-DiPT	Decreased freezing responses, higher potency in female mice.
Farinha-Ferreira et al., (80)	C57BL/6J mice	Psilocybin	Psilocybin increased HTR in both, acutely greater effect in females. Negative emotionality reduced; blocked by stress in males, only partially in females. Corticosterone in males - highest with stress + psilocybin, in females similar elevations across conditions.
Gattuso et al., (81)	Wild-type and SAPAP3 knockout mice	Psilocybin	Male WT mice: ↑ sociability; ↓ Lactobacillus (L. murinus/animalis) & Alistipes dispar (no broad diversity change). Female WT mice: no ↑ sociability; no comparable taxa changes. SAPAP3 KO mice: social deficits; no clear social rescue. Overall: chronic psilocybin dose-dependently ↑ gut transit time.
Lu et al. (preprint, 82)	C57BL/6J mice	Psilocybin	Male mice: robust acute HTR; little/no reliable 24-h behavioral effects. Female mice: robust acute HTR (higher); little/no reliable 24-h behavioral effects.

HTR, head twitch response; 5-MeODMT, 5-methoxy-N,N-dimethyltryptamine; DMT, N,N-Dimethyltryptamine; 4-OH-DiPT, 4-hydroxy-N,N-diisopropyltryptamine.

Summary of the available evidence of sex-related differences in the effects of psychedelics on behaviors associated with specific experimental paradigms designed to mimic disorders or behavioral conditions in animals. The table highlights the substance, species, and strain of the tested animals.

## 4 Discussion

This review aimed to determine whether sex differences in psychedelics research on rodents represent isolated, non-reproducible findings or whether they follow a systematic and analyzable pattern. The gathered evidence supports the existence of sex-dependent differences in the effects of psychedelics in rodents. Moreover, these differences appear to be not only influenced by biological sex alone but also by the ovarian cycle phase or behavioral mechanisms, highlighting many layers of variability in psychedelic responses.

The most well-documented evidence, based on the highest number of publications, concerns psilocybin and its metabolite psilocin (**Supplementary 4**). This compound is the second most frequently studied psychedelic in medical databases, following LSD (**Supplementary 4**). Both substances exhibit sex-dependent effects across nearly every examined category, except pharmacokinetics and conditional behaviors, for which there was insufficient literature. The most substantial number of documented sex differences occurs in unconditional behavior models, followed by model paradigms or neuroplasticity-related effects.

Although the presence of sex-dependent psychedelic effects is well-supported, more substantial evidence is required in the multiple domains. Only a single study mentioned in this review has directly examined the relationship between the ovarian cycle and the effects of LSD. While it is clear that psychedelics elicit different effects in males and females, the overall pattern of these differences is not straightforward. It is inaccurate to generalize that females are either more or less vulnerable to psychedelic effects; instead, the magnitude and direction of these differences depend on the specific parameter being assessed. This complexity is particularly evident across 5-HT2A-linked outcomes, where the direction and magnitude of sex differences vary depending on the compound, dose, strain, behavioral domain, and ovarian cycle phase.

The research compiled in this review indicates that female rodents display heightened responses to psychedelic compounds across multiple domains. Female mice exhibited lower brain concentrations of DOI (63), despite comparable or enhanced behavioral effects, increased spine formation in the frontal cortex (68), increased stimulus-specific CeA reactivity (13), greater reactivity to aversive stimuli (13), higher body temperature following psychedelic administration (21), a negative correlation between PPI and startle response (54), and increased head-twitch response (HTR) frequency (63). However, not all neurochemical effects followed the same female-biased pattern. In the 25I-NBOMe study, females showed higher dopamine levels in the medial prefrontal cortex, whereas males showed a larger drug-induced increase in extracellular serotonin in the same region (21). HTR frequency was similar in both males and females of the 129S6/SvEv mouse strain (63), suggesting that strain-specific factors may also influence certain aspects of the response (**Supplementary 3**). In this context, it is worth mentioning that the 129S6/SvEv strain generally displays lower exploratory activity in the plus-maze and locomotor activity tests (83).

Conversely, some psychedelic-induced outcomes were more prominent in male rodents, including reduced voluntary alcohol consumption after psilocybin administration (77), time-dependent decreases in central amygdala reactivity (13), a positive correlation between PPI and startle response (54), and greater sensitivity to psilocin-induced locomotor suppression, which has been attributed to differential activation of 5-HT1A and 5-HT2B/C receptors (14). These differences suggest that psychedelic effects are shaped not only by drug-receptor interactions but also by the underlying genetic, hormonal, metabolic, and neuronal environment.

The mechanistic basis of these sex differences may primarily involve two points of action: 5-HT receptor agonism and BDNF–TrkB signaling (31), the latter of which remains mechanistically debated (32). Activation of cortical 5-HT receptors initiates a cascade predominantly increasing glutamatergic activity in the prefrontal cortex, which subsequently influences dopaminergic, noradrenergic, serotonergic, and cholinergic systems through widespread network effects, leading to activity-dependent BDNF release and TrkB-mediated synaptogenesis. Interestingly, although females exhibit lower brain and plasma concentrations of DOI, they display higher HTR rates, suggesting that faster elimination does not diminish, and may even enhance, behavioral sensitivity. One possible explanation involves the concept of receptor reserve, whereby maximal functional responses are achieved despite submaximal receptor occupancy (63). However, this mechanism would predict increased 5-HT2A receptor expression in females, which has not yet been demonstrated. Sex-specific differences in downstream signaling efficiency or receptor coupling may therefore provide a more parsimonious explanation for the heightened behavioral sensitivity observed in females (63), consistent with evidence that psychedelics amplify extracellular neurotransmitter levels through recurrent glutamatergic activity in a manner that is not strictly dose-dependent (26). Thus, the female-biased HTR observed despite lower DOI concentrations is unlikely to be explained by drug exposure alone. Instead, it may reflect sex-specific differences in downstream 5-HT2A receptor coupling, signaling efficiency, or interactions with hormone-dependent serotonergic regulation.

Metabolic differences may further contribute to these sex-specific effects. Faster psychedelic metabolism in females may result from sex-divergent liver metabolism (84) or enzymatic transformation within the central nervous system. A comparable mechanism has been observed with psilocybin, which undergoes conversion to psilocin, a metabolite with increased lipid solubility that facilitates penetration of the blood-brain barrier. Consequently, psychedelic effects intensify following hepatic processing. A similar first-pass effect may also apply to other psychedelics, underscoring the importance of considering sex-based metabolic differences when interpreting psychedelic responses.

Across the studies we reviewed, the most consistent predictor of *whether* a sex difference appeared at all was estrous-cycle phase rather than sex per se, visible in LSD-induced PPI disruption (73), psilocin-induced locomotion (14), and the proestrus/diestrus split in psilocybin-induced HTR (74). This pattern points away from static sex-as-binary explanations and toward dynamic, hormone-state-dependent modulation of serotonergic signaling, for which the molecular substrate has been mapped in detail. Specifically, Shadani et al. report that 17-β estradiol (E2) enhances serotonergic neurotransmission through multiple mechanisms: increasing tryptophan hydroxylase (TPH) production, inhibiting the serotonin reuptake transporter (SERT), and suppressing monoamine oxidase A (MAO-A), which collectively prolong serotonin availability in the synaptic cleft (85). Another mechanism involves the expression of specific subtypes of receptors. Under the influence of E2, expression of 5-HT2AR increases, and expression of 5-HT1AR decreases. These alter the efficacy of psychedelics. Additionally, estradiol fluctuates throughout the ovarian cycle, dynamically altering serotonin receptor binding (85). Under the influence of estradiol, 5-HT2A receptor expression or binding appears to increase, while 5-HT1A receptor expression may decrease (85). This shift could enhance sensitivity to 5-HT2A-mediated psychedelic effects during high-estradiol phases of the ovarian cycle. Biegon et al. (1980) observed a significant increase in 5-HT2A receptor density during the proestrus phase, when estrogen levels peak, indicating that ovarian cycle-dependent receptor regulation may contribute to variations in psychedelic sensitivity (86).

Beyond its role in serotonergic signaling, estradiol also influences BDNF release via NMDA receptor activity (87). Since BDNF release is calcium-dependent, hormonal modulation of NMDA receptor function may further impact TrkB activation, contributing to sex-dependent psychedelic effects.

Given the substantial evidence for sex differences in psychedelic responses, therapeutic applications must account for these variations. In particular, women undergoing psychedelic-assisted treatment may show enhanced acute sensitivity during high-estradiol phases of the cycle (proestrus equivalent), reflecting estradiol-driven upregulation of 5-HT2AR expression and downstream plasticity-related signaling (including BDNF release, TrkB activation, and dendritic spine remodeling), a window in which therapeutic effects, but also adverse responses, may be amplified. Psychedelic drug metabolism, ovarian cycle-dependent effects, TrkB-mediated plasticity enhancement, and sex-specific gene expression changes are key factors that could influence treatment outcomes. A potential avenue for future research is the combination of psychedelics with neurosteroids such as allopregnanolone (Brexanolone), given their shared modulation of neuroplasticity-related pathways.

Human data add a social and experiential context to these preclinical findings (88, 89). Survey studies indicate that men are more likely than women to report lifetime psychedelic use, more frequent use, and higher doses, whereas women more frequently report adverse effects such as anxiety, paranoia, and confusion (90). These patterns are strongly shaped by social and cultural factors, but they are also broadly compatible with rodent evidence showing sex-dependent modulation of acute sensitivity, fear extinction, and stress-anxiety circuits (13, 63, 68, 74). Future preclinical and clinical studies should therefore include sex-stratified analyses, consider ovarian cycle or hormonal status where relevant, and use outcome measures able to detect both therapeutic and adverse responses. Reanalysis of existing datasets with sex as a covariate may also provide valuable insight into the mechanisms and translational relevance of sex-dependent psychedelic effects.

## 5 Limitations and methodological recommendations

Several limitations must be acknowledged. First, despite our expanded search strategy, the overall literature on sex differences in psychedelic effects remains sparse. Studies where sex effects appear in secondary analyses or supplementary materials may still have been missed, particularly for older publications. Second, the diversity of compounds, doses, routes, species, strains, and behavioral paradigms limits direct comparisons. Third, most studies lack systematic estrous-cycle tracking, which our review identifies as a critical gap.

Beyond limitations of the literature, the rodent model itself imposes fundamental constraints on what preclinical psychedelic research can address. Rodents cannot report the subjective phenomenology that is increasingly recognized as central to psychedelic therapeutics in humans: ego dissolution, mystical-type experience, and the personal insights that often emerge during clinical sessions. Equally absent are the contextual ingredients that clinical work treats as therapeutic in their own right, such as the carefully prepared set and setting, the structured therapist–patient relationship across preparation, dosing, and integration sessions, and the post-acute period in which patients verbally re-frame their experience and consolidate behavioral change through narrative integration. Long-term clinical outcomes in humans appear to depend on these processes; in rodents, where post-dose “integration” can only be operationalized as a passive return to the home cage, any persistent behavioral change must be attributed to neurobiological after-effects of the drug alone, without the psychological scaffolding that supports therapeutic outcomes in patients. This asymmetry constrains how strongly preclinical findings, including the sex-dependent effects synthesized here, can be projected onto clinical populations, and it should be read as a horizon, not as a reason to discount rodent work.

Based on our synthesis, we recommend that future preclinical psychedelic studies:

1. Include both sexes and analyze sex × treatment interactions as standard practice, rather than pooling data or reporting sex effects only when significant.
2. Track and report estrous-cycle phase, given its demonstrated ability to determine whether sex differences are detected.
3. Use multiple strains where feasible, given the demonstrated strain dependence of sex effects.
4. Extract and report sex-related data from supplementary analyses in published datasets, following the NIH mandate to consider sex as a biological variable.

## 6 Conclusions

Sex differences in the effects of psychedelics, including the influence of the ovarian cycle, have been confirmed in rodent models. These differences arise from complex interactions between serotonergic, neurotrophic, and metabolic pathways, with females generally exhibiting heightened sensitivity to psychedelics, though the extent of this sensitivity varies based on dose and hormonal state. While clear sex-dependent effects have been identified, gaps remain in pharmacokinetics, conditional behaviors, and the precise role of hormonal fluctuations.

Many previous studies have either focused solely on male subjects or combined data without accounting for sex-dependent variability, limiting the scope of current knowledge. Revisiting existing datasets and ensuring sex-specific analyses in future research are essential for a more comprehensive understanding of psychedelic action. Given the renewed clinical interest in psychedelics, these findings underscore the importance of integrating sex differences into both experimental design and therapeutic applications to optimize treatment efficacy and safety.
