# Supplementary material for: Sex-dependent effects of psychedelics: review of evidence from rodent models
Source: Front Psychiatry. 2026 Jul 15;17:1824073. doi: 10.3389/fpsyt.2026.1824073 (PMC13415512; doi:10.3389/fpsyt.2026.1824073)
Supplement: Supplementary file 1 [file Table1.docx]

**Supplement 1** Phrases and count of publications. Data from 27.01.2026.

| phrases | Publications total number | English language only | After removing duplicates |
| --- | --- | --- | --- |
| Ayahuasca | 739 | 721 |  |
| Dimethyltryptamine | 1,376 | 1,332 |  |
| Lysergic acid diethylamide | 5,909 | 5,053 |  |
| Mescaline | 1,396 | 1,202 |  |
| psilocybin | 2,900 | 2,678 |  |
| TOTAL | 12,320 | 10,986 | 10,263 |
