# Supplementary material for: Sex-dependent effects of psychedelics: review of evidence from rodent models
Source: Front Psychiatry. 2026 Jul 15;17:1824073. doi: 10.3389/fpsyt.2026.1824073 (PMC13415512; doi:10.3389/fpsyt.2026.1824073)
Supplement: Supplementary file 2 [file Table2.docx]

**Supplement 2.** Phrases used in the second round of filtering and count of publications. Data from 27.01.2026.

| phrases | Publications total number |
| --- | --- |
| Female | 161 |
| Male | 361 |
| Mice | 331 |
| Rat | 3956 |
| Gender | 47 |
| Sex | 119 |
| Sex-dependent | 5 |
| Sex differences | 22 |
| Sex-specific | 5 |
