# Supplementary material for: Sex-dependent effects of psychedelics: review of evidence from rodent models
Source: Front Psychiatry. 2026 Jul 15;17:1824073. doi: 10.3389/fpsyt.2026.1824073 (PMC13415512; doi:10.3389/fpsyt.2026.1824073)
Supplement: Supplementary file 3 [file Table3.docx]

**Supplement 3.** Rodent strains characteristic.

| Animal strain | Publication | Characteristics |
| --- | --- | --- |
| C57BL/6J mice | Jaster et al., 2022;  Shao et al., 2021; Biosca-Brull et al., 2024; Alper et al., 2023; Farinha-Ferreira et al., 2025;  Kelly et al., 2023 | Mice characteristic for their dark brown coat. Characteristic for low bone density. Used as “genetic background" for genetically modified mice for use as models of human disease. Widely used due to the availability of [congenic](https://en.wikipedia.org/wiki/Congenic) strains, easy breeding, and robustness. |
| 129S6/SvEv mice | Jaster et al., 2022; Vohra et al., 2022; Villalba et al., 2024 | Show more sociability, oftenly used in measuring locomotion, metabolism, exploratory behaviors. Oftenly used in genetic research and is also used as a popular “genetic background”. 129S6/SvEv strain generally display lower exploratory activity in the plus-maze and locomotor activity test. |
| Sprague–Dawley rats | Miliano et al., 2019;  Effinger et al., 2023; Dickinson and Curzon, 1986; Kennett et al., 1986; Cameron et al., 2019 | White albino rat. Known for calm demeanor, adaptability, and genetic reliability. |
| Long Evans rats | Roberts et al., 2023; Zylko et al., 2025 | Mix of albino Wistar and wild type Rattus norvegicus, characteristic for its black and white coat. Model resistant to oncogenesis. Widely used in behavioral, cognitive learning, ageing or addiction (especially alcohol addiction). |
| Wistar rats | Páleníček et al., 2010; Tylš et al., 2016 | Albino strain. The most popular rat strain used as experimental material in epidemiological research, [clinical trial](https://www.sciencedirect.com/topics/pharmacology-toxicology-and-pharmaceutical-science/clinical-trial), genetic study, or mechanism elaboration of pathway signaling. |
| Fawn Hooded rats | Meehan and Schechter, 1998 | Outbred stock that results from crosses of other outbred rats: German Brown, Lashley albino and  Long Evans. Exhibit hypertension leading to proteinuria and focal glomerular sclerosis. Show responsiveness of serotonergic mechanisms in the central nervous system, alcoholism and hypercortisolemia. |
|  |  |  |
