# Supplementary material for: Sex-dependent effects of psychedelics: review of evidence from rodent models
Source: Front Psychiatry. 2026 Jul 15;17:1824073. doi: 10.3389/fpsyt.2026.1824073 (PMC13415512; doi:10.3389/fpsyt.2026.1824073)
Supplement: Supplementary file 4 [file Table4.docx]

Supplement 4: Subsections and substances used in the study.

|  | **DMT**  **And 5-MeODMT** | **LSD** | **Psilocybin and psilocin** | **Ibogaine and noribogaine** | **DOI** | **Others (25I-NBOMe, PCA, Apomorphine, SKF-82.958, 4-OH-DiPT)** | **Total** |
| --- | --- | --- | --- | --- | --- | --- | --- |
| **Pharmacokinetics** |  |  |  |  | Jaster et al., 2022 |  | 1 |
| **Physiology and neuroplasticity** |  |  | Effinger et al., 2023  Shao et al., 2021 | Villalba et al., 2024  Biosca-Brull et al., 2024 |  | Miliano et al., 2019 (25I-NBOMe) | 5 |
| **Behavior** |  |  |  |  |  |  |  |
| **Unconditional behaviour** | Dickinson and Curzon, 1986 | Páleníček et al., 2010  Vohra et al., 2022 | Roberts et al., 2023  Tylš et al., 2016  Zylko et al., 2025 |  | Jaster et al., 2022  Vohra et al., 2022 | Dickinson and Curzon, 1986 (PCA)  Vohra et al., 2022 (Apomorphine and SKF-82.958) | 10 |
| **Conditional behaviour** |  | Meehan & Schechter, 1998 |  |  |  |  | 1 |
| **Model** | Kennett et al., 1986  Cameron et al., 2019 |  | Alper et al., 2023  Farinha-Ferreira et al., 2025  Gattuso et al., 2025 |  |  | Kelly et al., 2023 (4-OH-DiPT  ) | 6 |
| **Total** | 3 | 3 | 8 | 2 | 3 | 4 | 23 |
